# Construction, Spectral Modeling, Parameter Inversion-Based Calibration, and Application of an Echelle Spectrometer

**DOI:** 10.3390/s23146630

**Published:** 2023-07-24

**Authors:** Yuming Wang, Youshan Qu, Hui Zhao, Xuewu Fan

**Affiliations:** 1 Xi’an Institute of Optics and Precision Mechanics, Chinese Academy of Sciences, 17 Xinxi Road, Xi’an 710119, China; wangyuming2016@opt.cn (Y.W.); quyoushan@opt.ac.cn (Y.Q.); zhaohui@opt.ac.cn (H.Z.); 2 School of Optoelectronics, University of Chinese Academy of Sciences, Beijing 100049, China

**Keywords:** echelle spectrometer, genetic simulated annealing, parameter inversion, laser-induced breakdown spectroscopy, calibration

## Abstract

We have developed a compact, asymmetric three-channel echelle spectrometer with remarkable high-spectral resolution capabilities. In order to achieve the desired spectral resolution, we initially establish a theoretical spectral model based on the two-dimensional coordinates of spot positions corresponding to each wavelength. Next, we present an innovative and refined method for precisely calibrating echelle spectrometers through parameter inversion. Our analysis delves into the complexities of the nonlinear two-dimensional echelle spectrogram. We employ a variety of optimization techniques, such as grid exploration, simulated annealing, genetic algorithms, and genetic simulated annealing (GSA) algorithms, to accurately invert spectrogram parameters. Our proposed GSA algorithm synergistically integrates the strengths of global and local searches, thereby enhancing calibration accuracy. Compared to the conventional grid exploration method, GSA reduces the error function by 22.8%, convergence time by 2.16 times, and calibration accuracy by 7.05 times. Experimental validation involves calibrating a low-pressure mercury lamp, resulting in an average spectral accuracy error of 0.0257 nm after performing crucial parameter inversion. Furthermore, the echelle spectrometer undergoes a laser-induced breakdown spectroscopy experiment, demonstrating exceptional spectral resolution and sub-10 ns time-resolved capability. Overall, our research offers a comprehensive and efficient solution for constructing, modeling, calibrating, and applying echelle spectrometers, significantly enhancing calibration accuracy and efficiency. This work contributes to the advancement of spectrometry and opens up new possibilities for high-resolution spectral analysis across various research and industry domains.

## 1. Introduction

The spectrometer serves as a pivotal instrument for investigating the intricate interplay between light and matter [1,2]. Echelle spectrometers find widespread application in the realm of astronomy and related disciplines, enabling high-resolution spectroscopy. These instruments employ diffraction gratings and an array of prisms to disentangle light into its constituent wavelengths, facilitating the simultaneous direct acquisition of comprehensive, high-resolution spectral information without the need for scanning. Given these remarkable advantages, the echelle spectrometer stands as one of the most potent tools for delving into the mysteries of the cosmos [3,4,5,6,7].

The first crucial step in achieving high spectral resolution lies in the construction of an aberration-free echelle spectrometer. However, the conventional symmetric Czerny–Turner (C–T) type of echelle spectrometer encounters challenges in simultaneously meeting the requirements of broad spectrum coverage, high resolution, and superior imaging quality [8,9]. In recent years, numerous efforts have been made to overcome these limitations and eliminate the aberrations of the echelle spectrometer. For instance, an asymmetric structure has been developed, capable of eliminating coma at specific wavelengths under certain conditions [10]. Additionally, the utilization of free-form cylindrical lenses [11] or toroidal mirrors [12] has been employed to counteract spherical aberration. Notably, Wood and Lawler devised a method involving the rotation of auxiliary dispersive elements [13]. However, the aforementioned methods impose high demands on optical processing and assembly, resulting in high costs and long cycles, which are not conducive to product industrialization.

Another critical factor in achieving high spectral resolution lies in the processing of spectrograms. The extraction of vital information from echelle spectra necessitates the utilization of precise spectral inversion algorithms. In 1982, Moreno et al. proposed a digital image processing technique that automatically mitigates geometric distortions in echelle spectra [14]. In 2002, Piskunov et al. presented an algorithm that processes traditional and cross-dispersed echelle spectra and exhibits exceptional quality in terms of noise level and CCD defects [15]. In 2016, Yin et al. introduced a high-precision spectral reconstruction algorithm that established a one-to-one correspondence between the wavelength and pixel position [16]. The algorithm’s accuracy was improved by calculating the offset distance of the primary light beam from the center of the image plane in the two-dimensional vertical direction and by compensating for the spectral line curvature of the reflective prism. In 2018, Duan et al. presented a simple spectral denoising algorithm that combines the least-squares method with polynomial fitting, effectively addressing the minor variations in spectrometer parameters caused by environmental changes, thus boosting the accuracy of the spectrometer [17]. Finally, in 2019, Xu et al. proposed two algorithms for the correction and smoothing of continuous echelle spectra: Alpha-shape fitting to spectrum and alpha-shape and lab source fitting to spectrum for the correction and smoothing of continuous echelle spectrum [18].

Despite the importance of accurate spectral inversion, current methods for estimating system parameters in echelle spectrometers are constrained by certain limitations. Some techniques rely on assumptions that may not hold true in practical applications, whereas others may be susceptible to systematic errors. Consequently, there is a pressing need for innovative algorithms that can better estimate system parameters and improve the accuracy of spectral inversion. With the continuous promotion and wide application of genetic algorithms (GA) in the optimization, pattern recognition, and machine learning fields since the 1990s, GA has been combined with other optimization techniques, such as neural networks and simulated annealing (SA), to create hybrid algorithms that could solve more complex problems. In recent years, GA has continued to evolve and be used in increasingly complex and challenging problems in finance, engineering, and biology to optimize investment portfolios, efficiently design products, and simulate biological systems, respectively. In addition, GA has been integrated into machine-learning algorithms [19,20,21] to optimize the parameters of deep neural networks and achieve state-of-the-art performance in many tasks, such as computer vision and natural language processing.

This paper introduced a compact and asymmetric three-channel echelle spectrometer, exhibiting remarkable capabilities in high-spectral resolution. Due to the inevitable errors encountered during processing and assembly, we propose a novel spectral inversion algorithm specifically designed for echelle spectrometers, focusing on the precise estimation of system parameters. By integrating various optimization techniques, including the genetic simulated annealing algorithm (GSA), significant enhancements in calibration accuracy are achieved. Our algorithm surpasses existing methods by incorporating a robust optimization approach that effectively addresses uncertainties in the measured data. To validate the efficacy of our algorithm, we conduct experiments using both simulated and real-world echelle spectra. The results firmly establish the superiority of our algorithm in terms of spectral resolution and accuracy compared to existing methods. This breakthrough in spectral inversion represents a significant advancement for echelle spectrometers, holding wide-ranging implications for applications in astronomy, atmospheric science, and related fields. Furthermore, experimental validation showcases exceptional spectral resolution and time-resolved capability, emphasizing the practical significance of our research in scientific and industrial contexts. Overall, our novel echelle spectrometer, along with the spectral inversion algorithm and improved calibration accuracy, offers a valuable tool for precise spectral analysis, thereby paving the way for advancements in various domains.

## 2. Theory and Methods

### 2.1. Echelle Grating

**Figure 1:**
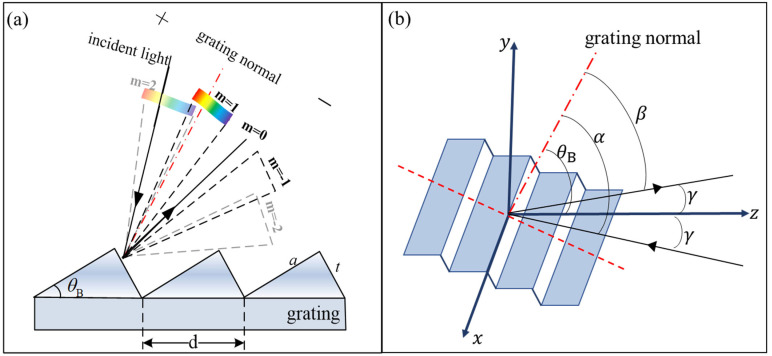
(**a**) 2D and (**b**) 3D schematic of the echelle grating.

The characteristics of echelle grating include a high diffraction order (from tens to hundreds), low groove density (several tens of lines per millimeter), large blaze angle (from 30° to 75°), full-band blaze (bandwidth covers deep ultraviolet to long-wave infrared), and low polarization effect. Owing to their good spectral performance and optical stability over the entire wavelength range, echelle gratings are widely used in various spectroscopic measurement systems, enabling instruments to be compact and achieve a high spectral resolution. For traditional gratings, the longer groove facets (length “*a*”) are oriented towards the incident and diffracted light, while for echelle gratings, the shorter and steeper groove facets (length “*t*”) are oriented towards the light, as shown in Figure 1. Figure 1a shows the working process of the reflective echelle grating dispersion. Polychromatic light is incident at a certain angle and is diffracted by the echelle grating to the center (zero order) and both sides (higher orders). The diffraction intensity and efficiency of a medium-level grating can be changed by tilting the grating. When the echelle grating was tilted to its blaze angle $\theta_{B}$, the diffraction efficiency was the highest, and most of the light in the target band was diffracted to the corresponding orders. However, the higher the order, the greater the overlap between adjacent orders, which may interfere with the extraction and use of the target band.

### 2.2. Cross-Dispersion

**Figure 2:**
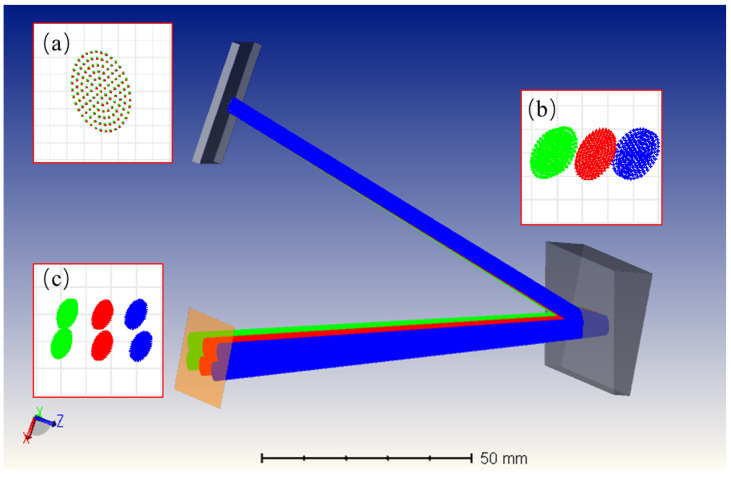
Schematic diagram of the principle of cross-dispersion. (**a**) Incident polychromatic light, (**b**) Light subjected to dispersion by a diffraction grating. (**c**) Light after undergoing cross-dispersion.

Cross-dispersion refers to the two-dimensional spectral dispersion achieved by passing light through two dispersion directions perpendicular to each other. Figure 2 illustrates the principle of cross-dispersion using the "grating + prism" configuration for six wavelengths. The point column diagrams of the grating, prism, and detector surfaces are shown in Figure 2a, b and c, respectively. After passing through the grating, the polychromatic light achieved good dispersion in a single order but with severe overlap between orders. However, after cross-dispersion using a prism, the spacing between orders significantly increased, improving the resolving power of the instrument. Cross-dispersion elements also called “auxiliary dispersion elements” are commonly used with planar gratings or prisms. The best cross-dispersion effect was achieved by matching different types of gratings and prisms. In addition, some researchers have used a dual-pass system to improve the resolution and throughput further [22]. However, cross-dispersion also has limitations, one of which is its sensitivity to placement errors, causing significant performance degradation. Automatic collimators and other alignment techniques are required to overcome these limitations.

### 2.3. Optical Design

**Figure 3:**
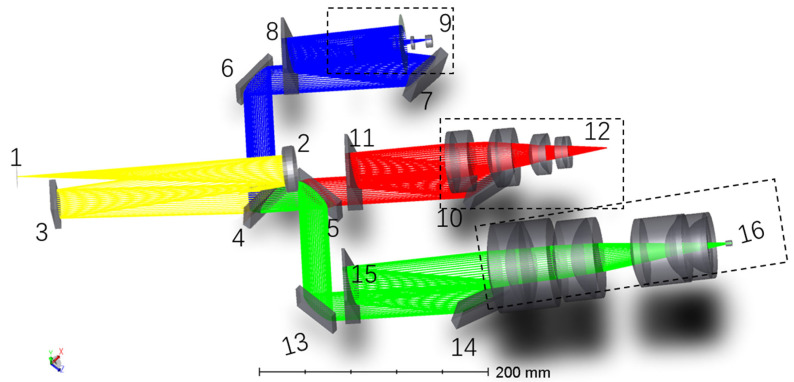
Optical path through the designed spectrometer. 1: slit; 2: off-axis parabolic collimating mirror; 3, 6, and 10: folding mirror; 4 and 5: beam splitter; 7, 11, and 14: echelle grating; 8, 12, and 15: dispersive prism; 9, 13, and 16: camera.

We accomplished an optical design to correct aberrations in an echelle spectrometer with an ultrawide spectral range. For detailed information, refer to [23]. The overall structure of the optical system is shown in Figure 3. The measured signal enters the system through a slit (1), is reflected by an off-axis parabolic mirror (2), and collimates into parallel light. The parallel light undergoes three-stage splitting after being reflected by a folding mirror (3) to lay out the optical path properly. First-stage splitting was achieved using two beam splitters (4 and 5). Beam splitter 1 (4) has a front surface coated with a multilayer color separation film that reflects all light within the 200–450 nm wavelength range and reflects part of the light in the 450–500 nm wavelength range to channel 1. All other wavelength ranges (500–1100 nm) are transmitted to beam splitter 2 (5). The front surface of beam splitter 2 is also coated with a multilayer color separation film, which reflects the remaining light in the 450–500 nm wavelength range, all light within the 500–800 nm wavelength range, and part of the light within the 800–850 nm wavelength range to channel 2; the remaining light (the remaining part within the 800–850 nm range and all light within the 850–1100 nm range) is transmitted to channel 3. Elements 6 and 10 are flat mirrors for channels 1 and 2, respectively, and they make the cross-dispersed structures of the three channels parallel, facilitating mechanical processing and practical operations. The second- and third-stage splitting is completed by three medium-grade step gratings (7, 11, and 14) and dispersing prisms (8, 12, and 15) in each channel. After three-stage splitting, the light enters the designed focusing mirrors (9, 13, 16) with different two-dimensional fields of view.

### 2.4. Spectral Model

#### 2.4.1. Establishment of Theoretical Two-Dimensional Spectrum

For a given diffraction order *m* and grating constant *d*, the grating equation for any wavelength with an incidence angle *α* and diffraction angle β can be expressed as

(1) mλ=dsin⁡αcos⁡γ+dsin⁡βcos⁡γ

where *γ* denotes the grating deflection angle, which is the angle between the incident light and the principal plane, as shown in Figure 1b.

When the quasi-Littrow condition is satisfied, the incidence angle of the grating is equal to the blaze angle $\theta_{B}$; hence,

(2) $$\beta_{grating}(\lambda )=\arcsin\left\{\frac{m\lambda }{d\cos\gamma }-\sin\theta_{B}\right\}$$

Consequently, the relative coordinates in the main dispersion direction of the wavelength on the detector with respect to the center wavelength $\lambda_{cen}$ of the corresponding order can be calculated as follows:

(3) $$x(\lambda )=f_{foc}\times \tan(\beta_{grating}(\lambda )-\theta_{B})$$

where $f_{foc}$ is the camera focal length.

The relationship between the prism exit angle $\beta_{prism}(\lambda )$ and the wavelength λ is described as follows:

(4) $$\beta_{prism}(\lambda )=\arcsin\left\{n(\lambda )\sin\left\{2\rho -\arcsin\left(\frac{\sin i}{n(\lambda )}\right)\right\}\right\}$$

where i is the prism incident angle, n(λ) is the refractive index of the material corresponding to λ, and ρ is the prism apex angle.

Therefore, the absolute coordinates of λ corresponding to the cross-dispersion direction on the detector are given by

(5) $$y(\lambda )=f_{foc}\times \tan(\beta_{prism}(\lambda ))$$

The spectral reconstruction algorithms used in all three channels of the echelle spectrograph described in this study were similar. Therefore, the following analysis focuses on channel 1, which has the most covered orders, the strongest nonlinearity, and the densest and most complex spectral lines of the featured elements.

**Figure 4:**
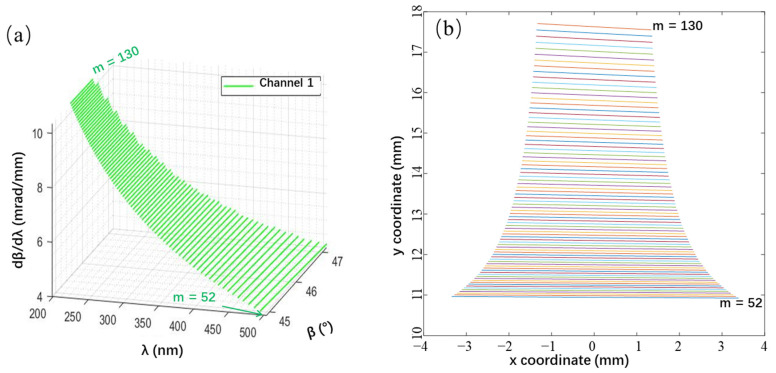
Theoretical Two-Dimensional Spectrum of the designed spectrometer. (**a**) Wavelength–diffraction angle–angular dispersion relationship of the echelle gratings. (**b**) Spectral model coordinates.

Based on the calculated results of the two-dimensional coordinates corresponding to different wavelengths, we obtain the theoretical two-dimensional spectrum, as shown in Figure 4, which confirms that (1) the spacing between orders will decrease in the long-wavelength direction owing to the decrease in the prism’s refractive index variation; (2) as the wavelength increases, the order gradually decreases, but the free spectral range becomes longer.

#### 2.4.2. Prototype Construction and Data Acquisition

**Figure 5:**
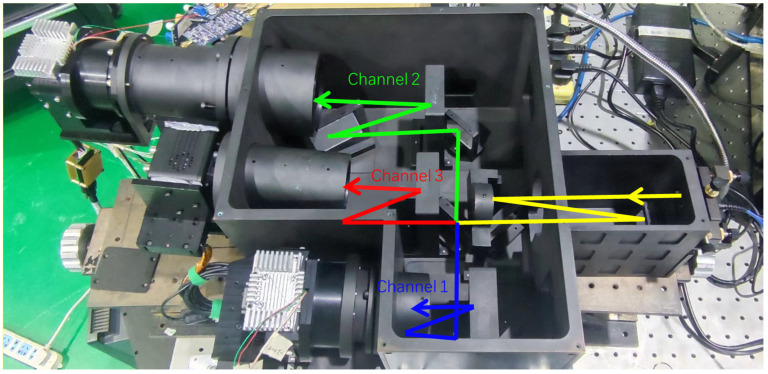
Prototype of the Echelle spectrometer.

The echelle spectrometer was processed and assembled according to the optical design scheme; the principal prototype of the assembled device is shown in Figure 5. This spectrometer with overall dimensions of 600 × 280 × 130 mm has the advantages of small space occupation, compact structure, high adaptability to temperature variations, and high mechanical stability. The compact structure makes it easy to carry and install in practical applications; moreover, due to the utilization of Invar as the structural material, along with the incorporation of fused quartz as the optical material, both renowned for their low thermal expansion coefficients, the instrument exhibits remarkable versatility in accommodating temperature variations. Consequently, it enables precise measurements not only within controlled laboratory environments but also in demanding field conditions with specific temperature requirements. In addition, its high mechanical stability ensures measurement accuracy and stability. However, multiple light channels can produce stray light after separation, which may interfere with the adjacent channels of spectral measurements. Therefore, blackened light-blocking plates were installed between adjacent channels to prevent stray light from interfering with the spectral measurements.

**Figure 6:**
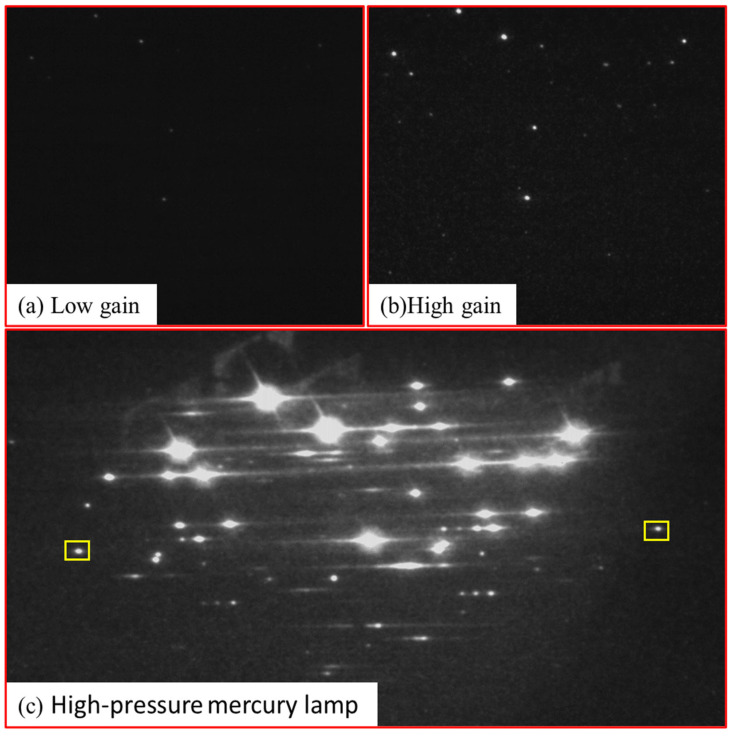
(**a**) Raw image of a low-pressure mercury lamp with low gain. (**b**) Raw image of a low-pressure mercury lamp with high gain. (**c**) Raw image of a high-pressure mercury lamp.

The echelle spectrograph was used to capture the image of the low-pressure mercury calibration lamp; the original image of channel 1 is shown in Figure 6. The spectrum has the following characteristics:

(1) Large dynamic range. Although the effective spectral line width of the mercury lamp is very limited compared to the instrument’s detection range, the intensity differences are very distinct. In addition, the nonlinear enhancement effect of the amplifier on the spectral intensity makes it difficult to obtain a large amount of characteristic spectral line information through a single exposure.
(2) Complex spot diffusion: Although the optical design, processing, and adjustment ensure that the size of the diffraction spot corresponding to a single spectral line is within one pixel after merging, the characteristics of the amplifier can also affect spot diffusion. When the integration time × gain is high, the overexposed energy spreads to adjacent pixels, causing interference and crossover between pixels, significantly affecting the spectral resolution.
(3) Complex background: Owing to the use of the amplifier and detector, the noise and redundant information in the image are very complex, including gain noise caused by the unstable amplifier gain and thermal, environmental, and dark current noise of the detector. In particular, when the integration time × gain is relatively high, some weak signals will be submerged in the noise signal, decreasing instrument sensitivity.
(4) Particular wavelengths spots may “leapfrog to the next diffraction order”: In an ideal echelle spectrometer, each stripe of the spectrum exclusively contains the free spectral range of its corresponding order, whereas other wavelengths near the boundary of that order converge onto neighboring orders. Nonetheless, in practice, certain transitional wavelengths may manifest simultaneously in two adjacent orders, as illustrated by the highlighted yellow area in Figure 6c, significantly reducing the precision of the spectral model correction.

## 3. Data Processing and Calibration Methods

### 3.1. Data Pre-Processing Methods

The aforementioned analysis indicates that to calibrate an echelle spectrometer accurately, we refer to the wavelength-intensity image of the calibration lamp and correct the actual two-dimensional image of the detector to obtain an accurate spectral model. The primary methods to achieve these are as follows:

(1) Multiple exposures: In the case of significant differences in the intensity of characteristic spectral lines, a multiple-exposure strategy with different integration times × gain multiples can be employed. At low integration times × gain multiples, the signal-to-noise ratio in the image remains high and could be selectively extracted as a calibration reference, as shown in Figure 6a. Weak signals can be extracted from the image at high integration times × gain multiples, as shown in Figure 6b. This strategy can effectively improve the detection sensitivity for samples with significant signal intensity differences without sacrificing the signal-to-noise ratio. Notably, excessively high integration times × gain multiples may severely affect the spectral resolution by forming overexposed bright spots, as shown in Figure 6c.
(2) Background removal and filtering: Background removal methods include linear and nonlinear background fitting to remove background noise effectively while preserving signal morphology. Filtering methods include low-pass, high-pass, bandpass, and bandstop filtering, which filters noise within specific frequency ranges. These preprocessing methods aim to eliminate background noise and clutter in the signal and improve the signal-to-noise ratio and accuracy, thus increasing the reliability and accuracy of spectral analysis [24].
(3) Centroid extraction: In pixels with severe diffusion or surrounding background noise, centroid extraction algorithms [25] can locate the wavelength by calculating the weighted average signal intensity. Signal information from multiple pixels can be used to improve the wavelength inversion accuracy and avoid calibration errors.
(4) Model correction: The model must be corrected by introducing an error evaluation function and using multiple wavelengths for a comprehensive evaluation to address the problem of edge wavelengths potentially drifting to adjacent orders. The key parameters that best fit the actual situation can be inverted, and a more accurate spectral model can be established.

Centroid extraction algorithms and model corrections are essential when reconstructing an echelle spectrometer spectrum. This is due to the intricate nature of spectral signals and the erratic noise distribution, making spectral peak fitting particularly prone to error. If the determination of the diffraction order is incorrect, it could result in an unacceptable range of wavelength errors, from 2 to 30 nm. Therefore, precise centroid extraction algorithms and effective model correction methods are required to ensure accurate spectral restoration. Moreover, noise interference and suboptimal peak fitting can be mitigated to restore and extract the original spectral signal. Because centroid extraction algorithms are well established, this study focuses on the precise inversion of the echelle grating nonlinear two-dimensional spectrum model.

### 3.2. Parametric Inversion Method

The parametric inversion method is a calibration technique used for echelle spectrometers. It aims to determine the relationship between the pixel position on the detector and the corresponding wavelength by modeling the dispersion properties of the instrument.

In traditional echelle spectrometer calibration, a set of known spectral lines or reference sources is used to establish a calibration curve. However, this method may not capture all the complexities and non-linearities of the system, leading to inaccuracies in the calibration. The parametric inversion method takes a different approach by using a physical model to describe the dispersion properties of the echelle spectrometer. Instead of relying solely on reference sources, it uses a theoretical model that incorporates the system parameters and physical properties of the instrument.

The parameter inversion results mentioned in this paper are all based on the two most sensitive parameters to spectral variations of the echelle spectrometer: the grating’s incident angle *α* and the deflection angle *γ*.

#### 3.2.1. Grid Search 

This simple and straightforward optimization algorithm involves systematically searching over a predefined set of parameter values. The algorithm updates one parameter at a time while keeping the others fixed and repeats the process until it covers the entire search space. A grid search is easy to implement; however, it is computationally expensive and need not converge to a global optimum.

#### 3.2.2. Simulated Annealing Algorithm 

**Figure 7:**
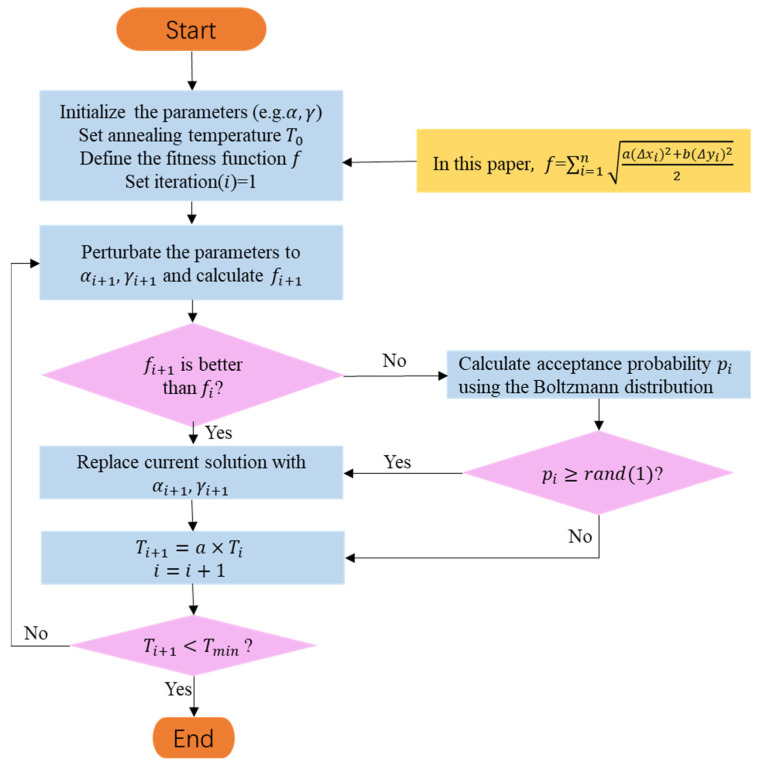
Flowchart of the SA algorithm.

SA is a global optimization algorithm inspired by the annealing process in physics. The annealing process searches for the global minimum of the cost function that minimizes the crystal structure defects of the material by slowly cooling it [26,27,28]. The obtained global minimum of the cost or objective function represents the system performance or solution quality. The SA algorithm is commonly used when conventional methods fail to efficiently solve the optimization problems. The flowchart for applying the SA algorithm to the parameter inversion of an echelle spectrometer is shown in Figure 7. The basic steps are as follows:

(1) Initialization: Establish the physical model of the echelle spectrometer and define the objective function based on design parameters and measured spectral data. The initial temperature is set to a high value, and an initial solution is randomly generated.
(2) Select a neighborhood: Randomly select a neighborhood solution near the current solution. In the case of an echelle spectrometer, the neighborhood solution can be generated by fine-tuning parameters such as grating constants, blaze angles, and grating incident angles.
(3) Calculate energy difference ΔE: Substitute the neighborhood solution into the objective function (such as the difference between the theoretical coordinates of the standard light source and actual coordinates of the spectral model) to calculate ΔE.
(4) Determine the acceptance probability: The acceptance probability is calculated based on the current temperature and ΔE. If the probability is greater than a random number, accept the neighborhood solution; otherwise, maintain the current solution.
(5) Cooling: The temperature is reduced using a preset cooling strategy (such as exponential or linear cooling).
(6) Determine the termination condition: If the termination condition is met (e.g., the temperature is lower than a certain set value or the algorithm has iterated sufficiently), the algorithm ends and returns the optimal solution; otherwise, return to Step 2.

In Step 4, the SA algorithm uses the Metropolis criterion [29] to ensure that the algorithm accepts inferior solutions with a certain probability, thereby avoiding becoming stuck in local optimal solutions. It also explains why the SA algorithm can achieve good results in solving complex problems.

The SA algorithm is highly sensitive to the initial temperature $T_{0}$. A higher initial temperature considers a wider solution space but increases the risk of falling into local minima. Although the algorithm is effective, it is computationally expensive and time-consuming when dealing with large-scale and complex optimization problems. To avoid data overfitting, SA requires multiple iterations and appropriate stopping criteria to balance computational costs and desired accuracy. In the parameter inversion of an echelle spectrometer, the success of SA lies in selecting an appropriate cost function (also known as the fitness function) and cooling schedule to achieve a good optimization convergence rate.

#### 3.2.3. Genetic Algorithm 

GA can be traced back to the mid-20th century; the first systematic exposition of GA is the work of John Holland, a computer science professor at the University of Michigan. In his book “Adaptation in Natural and Artificial Systems”, published in 1975 [30,31], Holland described how computer algorithms can simulate the process of natural selection and evolution to solve optimization problems. He defined GA as search algorithms that use principles from genetics and natural selection to find solutions to problems.

The GA consists of three key steps: selection, crossover, and mutation. In the selection process, individuals with higher fitness values are selected as parents to generate offspring through a crossover. The crossover operator randomly selects one or more crossover points on the parent chromosomes and exchanges genetic information beyond these points to create new offspring chromosomes. This step is crucial for maintaining the genetic diversity and evolution of the population. However, the selection process incorporates randomness to avoid completely discarding low-fitness chromosomes.

**Figure 8:**
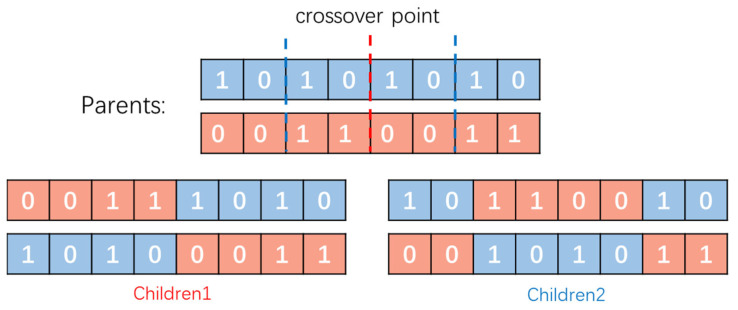
Crossover process in GA algorithms.

The schematic diagram of the crossover process in Figure 8 illustrates how a pair of parent chromosomes exchange genetic information to produce two offspring chromosomes. Each binary string represents a chromosome, and each digit represents a gene. The crossover points are shown in red and blue; the offspring’s chromosomes are given at the bottom.

**Figure 9:**
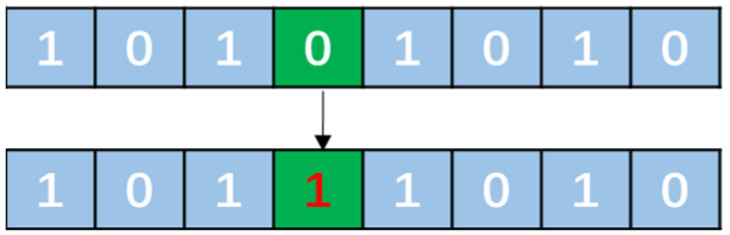
Schematic of the mutation process in GA algorithms.

Mutations are random processes that modify one or more genes on a chromosome and introduce new genetic information into the population. The mutation operator can swap gene positions or randomly execute genes to generate a new offspring. The number and intensity of the mutations can be controlled using the mutation probability and coefficient. A schematic diagram of the mutation operation in Figure 9 shows how a single gene in a chromosome is flipped from 0 to 1 or vice versa.

In the calibration of an echelle spectrograph using a genetic algorithm, the mutation operation involves randomly modifying the parameters of candidate calibration solutions. It serves several purposes: exploring the solution space, escaping local optima, maintaining genetic diversity, and fine-tuning solutions. With a high-dimensional parameter space, mutations allow for a broader search range, preventing the algorithm from getting stuck in local optima and facilitating the discovery of better calibration configurations. By introducing random variations, mutations help maintain genetic diversity among candidate solutions, enabling a more comprehensive exploration. Furthermore, mutations contribute to the refinement and improvement of solutions through small random modifications. Ultimately, the mutation operation plays a crucial role in optimizing the calibration process, enhancing the accuracy of the echelle spectrograph’s measurements.

**Figure 10:**
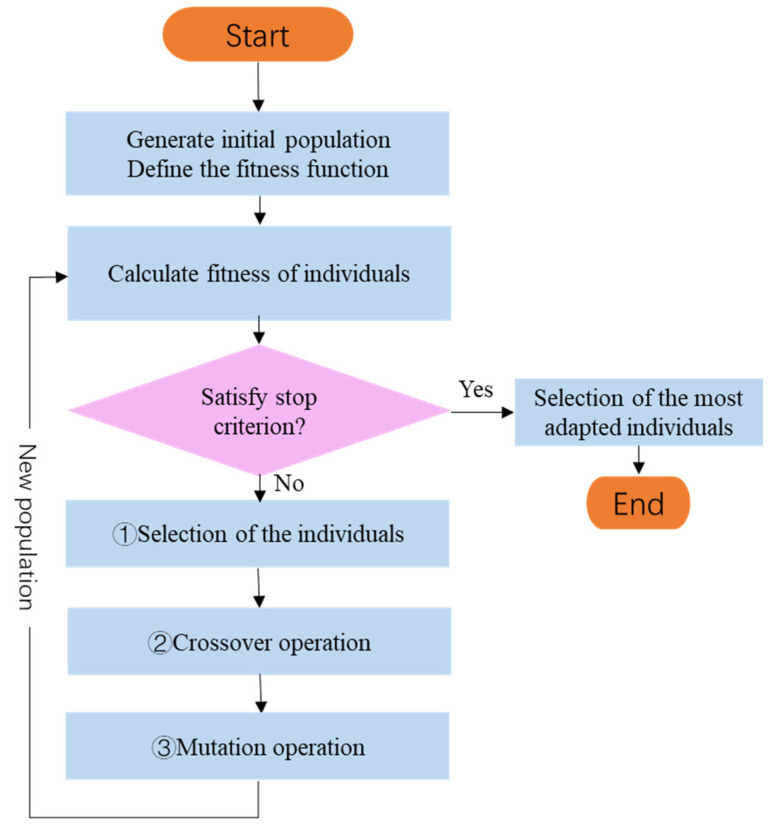
Flowchart of the GA algorithm.

Offspring chromosomes generated by these genetic operators were evaluated and replaced with low-fitness chromosomes in the population. The process is repeated until satisfactory solutions are obtained, or the stopping criteria are satisfied. The flowchart of the GA in Figure 10 summarizes the entire GA process, including the selection, crossover, and mutation operations, and replacing low- with high-fitness chromosomes.

GA can obtain solutions close to the global optimum for complex problems with no or insufficient mathematical expressions. However, complex high-dimensional multimodal problems require complex fitness functions with extensive computational time to precisely evaluate the optimal solutions. Therefore, effective methods, such as hyperparameter tuning and parallel processing, are often employed to search for solutions in large search spaces [32]. 

#### 3.2.4. Genetic Simulated Annealing Algorithm 

**Figure 11:**
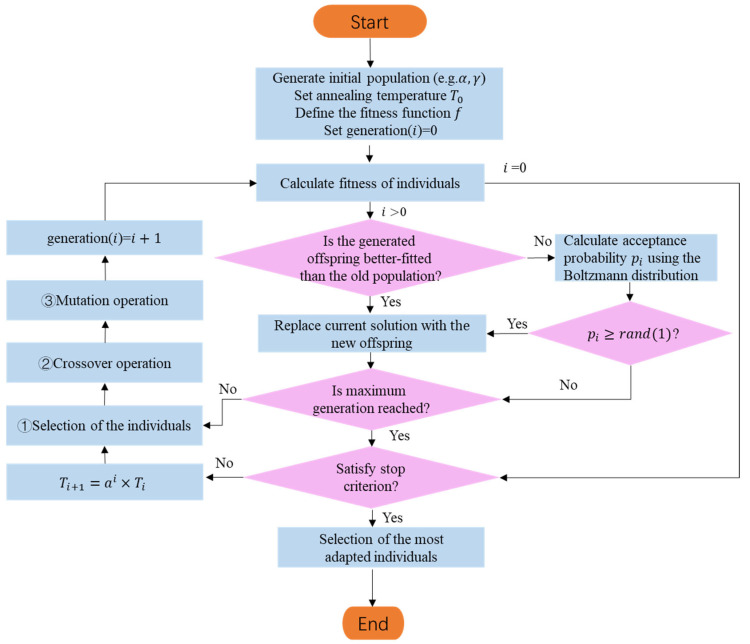
Flowchart of the genetic simulated annealing algorithm.

Given the maturity of the aforementioned algorithms and computer technology, coupled with the characteristics of the echelle spectrometer, this study models the calibration problem of the echelle spectrometer as a multi-objective optimization problem and proposes a solution based on GSA algorithm. This algorithm combines the advantages of the global search of GA and local optimization ability of the SA algorithm to efficiently search for the optimal parameter combination for the echelle spectrometer. Calibration of the echelle spectrometer parameters using GSA algorithm is illustrated in Figure 11, with the following basic steps.

(1) Population initialization: A group of candidate solutions is randomly initialized within a certain parameter range, with each chromosome representing a possible calibration parameter combination for the echelle spectrometer (such as a combination of the grating incident and deflection angles).
(2) Fitness evaluation: The coordinate reference set of known spectral lines on the detector is compared with the predicted spectral line coordinate set of each chromosome. A fitness function is set, and the matching degree of each individual is evaluated.
(3) Genetic operations: Genetic operations (such as mutation, crossover, and selection) are performed on individuals; the candidate solution population is evolved over multiple generations, and a new offspring population is generated.
(4) Reduce the mutation rate: The mutation rate is gradually reduced to promote the algorithm’s convergence toward a better solution.
(5) SA: The SA algorithm is periodically introduced to introduce randomness into the GA search process, help the algorithm jump out of local optimal solutions, and find better solutions.
(6) Check for termination conditions: If a satisfactory solution is found before reaching the maximum number of generations, the algorithm is stopped, and the best solution found is output.
(7) Maximum number of generations reached: If the maximum number of generations is reached without obtaining a satisfactory solution, the algorithm is stopped, and the best solution found is returned.
(8) Apply the best solution: The best result is used to adjust the corresponding parameters of the echelle spectrometer, a corrected two-dimensional spectrum is established, and it is used as a basis for judging other spectral wavelengths to improve calibration accuracy.

Compared with other optimization algorithms, GSA algorithm has several advantages in the calibration of echelle spectrometers. First, it is very effective in determining the global optimal solution of the objective function because it combines the global search ability of GA and the local search ability of SA. Second, the algorithm is robust and can handle complex and noisy data. Third, the algorithm is highly scalable and can be extended to optimize several parameters simultaneously.

## 4. Results

According to the above analysis, this study used the grid search method, SA, GA, and GSA algorithms to calculate the evaluation function values corresponding to different grating incidence angles and deflection angle errors.

### 4.1. Evaluation Function Determination

The adaptability function, also known as the evaluation function, is the first step in evaluating the quality of an evaluation algorithm. It serves as the foundation for analyzing the algorithm performance and determining the optimization direction. The evaluation function should accurately reflect the performance of the algorithm by avoiding overestimated evaluations or those restricted to specific datasets. Therefore, the diversity of the data and the comprehensiveness and objectivity of the evaluation indicators need to be considered when determining the evaluation function.

**Figure 12:**
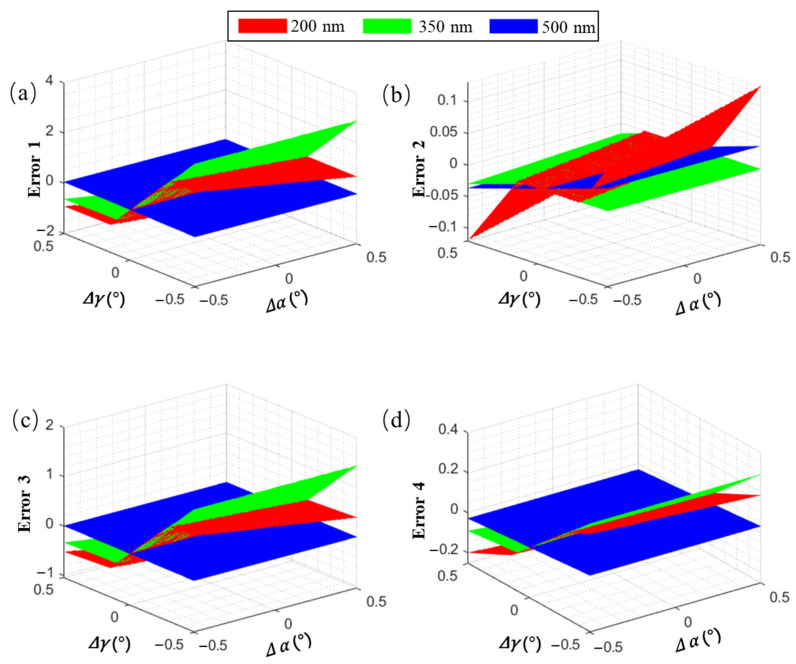
Relationship between the grating incidence angle deviation, deflection angle deviation, and different calibration error evaluation indicators. (**a**) Error 1: Δx, (**b**) Error 2: Δy, (**c**) Error 3:$\sqrt{\frac{{\Delta x}^{2}+{\Delta y}^{2}}{2}}$, (**d**) Error 4: $\sqrt{\frac{{a(\Delta x)}^{2}+{b(\Delta y)}^{2}}{2}}$, Δx and Δy respectively signify the disparities between the factual x and y coordinates of the wavelength on the detector and their theoretical counterparts.

For this study, the central wavelength of channel 1 at 350 nm and the upper- and lower-edge wavelengths at 200 and 500 nm, respectively, were selected. We selected the x- and y-coordinate deviations, Euclidean distance [33], and an improved deviation function as evaluation indicators for the detector spectrum coordinate deviation caused by grating incident and deflection angle deviations, which are the most sensitive angles for echelle spectrometers. The results are given in Figure 12.

In Figure 12, under the same error conditions, the difference between the x- and y-coordinates of a certain wavelength on the detector of the echelle spectrometer is significant; the longer the wavelength, the more the error tends to exhibit linear changes. In contrast, shorter wavelengths have a higher chance of exhibiting sudden changes in the error. The reason is that when the error is small, the wavelengths in the middle of each level will shift between levels, and only the edge wavelengths may experience “cross-level” phenomena. Therefore, the y-coordinate offset of most wavelengths is small, and the x-coordinate offset changes steadily. However, when the error accumulates to a certain degree, and the “leapfrog order” phenomenon occurs, the y-coordinate experiences a sudden change, and the x-coordinate changes from the maximum/minimum value to the minimum/maximum value.

Furthermore, the x-coordinate is highly sensitive to changes, with a range of −2–4 mm, while the range of the y-coordinate is only −0.1–0.1 mm. Therefore, a “Euclidean distance” evaluation function similar to that in Figure 12c cannot comprehensively measure the error level in the x and y directions. This study adopted an evaluation function similar to that shown in Figure 12d, which flexibly adjusts the weights of the *x* and *y* errors based on changes in the wavelength, making the calibration data more accurate.

Because we used multiple calibration light sources, such as mercury lamps and lasers, with multiple characteristic spectral lines, the evaluation function for n spectral lines can be set based on the above evaluation function $\sum_{i=1}^{n}\sqrt{\frac{a{\left(\Delta x_{i}\right)}^{2}+b{\left(\Delta y_{i}\right)}^{2}}{2}}$, which comprehensively evaluates the total error level for all characteristic wavelengths.

### 4.2. Optimization Results of Different Algorithms and Comparison

**Figure 13:**
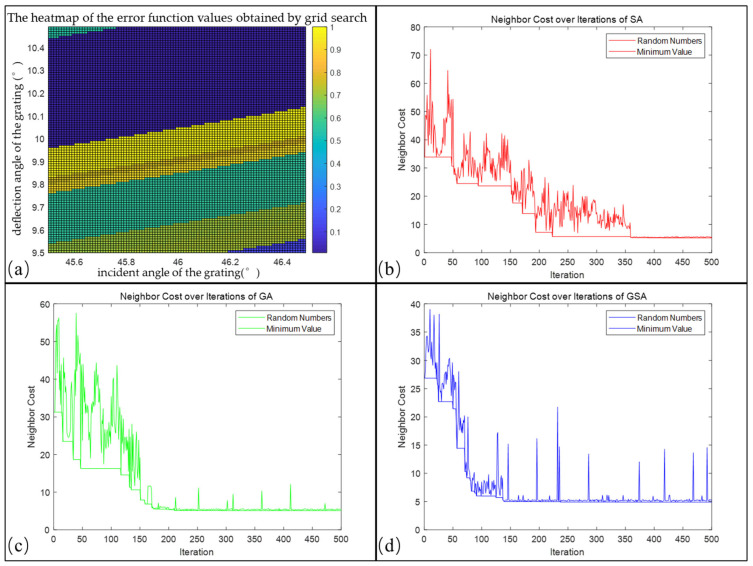
Optimization results of 4 different algorithms. (**a**) Heatmap of the error function values obtained by grid search, (**b**) graph of the number of iterations versus the evaluation function in the SA algorithm, (**c**) graph of the number of iterations versus the evaluation function in the GA algorithm, and (**d**) graph of the number of iterations versus the evaluation function in the GSA algorithm.

For the grid search method, the grating incidence and deflection angle errors were discretized and divided into a 100 × 100 matrix. Specifically, the considered grating incidence angle range was 45.5–46.5°, and the deflection angle range was 9.5–10.5°. Every possible combination of the grating incidence and deflection angle errors was calculated within this range, and the corresponding error function value was obtained. The results are shown in Figure 13a; the different colors represent different error values for each grid. Within this range, the error function exhibited a “layered” effect. This is because once the spectrum experiences “jumping”, the error will exceed a certain range, and the error function value will suddenly change. The method takes 1853 s to run, and the optimal combination of the grating incidence and deflection angles is (45.84° and 10.24°, respectively), with a minimum error value $\sum_{i=1}^{n}\sqrt{\frac{a{\left(\Delta x_{i}\right)}^{2}+b{\left(\Delta y_{i}\right)}^{2}}{2}}$ of 6.443.

For the SA algorithm, we set the initial temperature $T_{0}=1000$, cooling factor α=0.97, and the number of iterations to 500. It is important to note that the initial temperature and cooling factor are internal parameters of the algorithm that simulate the annealing process in metallurgy. These values are not directly associated with the physical temperature of the spectrometer. Typically, it is advisable to set a higher initial temperature to ensure that the obtained results are closer to the global optimum [34]. We recorded the value of the error function obtained at each iteration and displayed it, along with the minimum error value obtained during the iteration process, as shown in Figure 13b. As the number of iterations increases, the error function gradually approaches its minimum value. However, some fluctuations exist during the iteration process. In the spectral model calibration process, the error function of the SA algorithm converged to its minimum value (46.170°, 10.225°) after 361 iterations with an associated fitness function value $\sum_{i=1}^{n}\sqrt{\frac{a{\left(\Delta x_{i}\right)}^{2}+b{\left(\Delta y_{i}\right)}^{2}}{2}}$ of 5.287. The entire iteration process required 756 s, and the time required for convergence was 546 s.

GA was initialized with a population size of 50, mutation probability of 0.1, and maximum iteration of 500. During each iteration, the algorithm performs selection, crossover, and mutation operations on the population to produce an improved offspring. The best fitness value for each iteration is recorded and plotted against the iteration number, as shown in Figure 13c. The fitness value gradually decreases with increasing iterations; however, some fluctuations exist during the process, particularly after reaching the optimal solution. This is because the mutation process continues to explore better solutions beyond the local optima. Finally, the GA converges to local optimal solutions of (46.339° and 10.217°) after 211 iterations with a fitness value $\sum_{i=1}^{n}\sqrt{\frac{a{\left(\Delta x_{i}\right)}^{2}+b{\left(\Delta y_{i}\right)}^{2}}{2}}$ of 5.121. The overall running time of the algorithm was recorded as 1578 s, with a convergence time of 666 s.

GSA is an optimization method that combines two algorithms. It is similar to GA in population selection, crossover, and mutation; it incorporates the local search of the SA algorithm to find better solutions in the local range of the solution space. In addition, better results were obtained by adjusting the GA parameters to adapt to the problem characteristics. Therefore, we used the parameters of GA and introduced SA in GSA. The fitness function value of the best solution obtained in each iteration and the best value in the iteration process were recorded, as shown in Figure 13d. In the spectral model calibration process, the GSA finally converged to a local optimal solution at the 139th iteration, which corresponded to (46.2317°, 10.1775°), with a fitness function value $\sum_{i=1}^{n}\sqrt{\frac{a{\left(\Delta x_{i}\right)}^{2}+b{\left(\Delta y_{i}\right)}^{2}}{2}}$ of 4.977. However, owing to the complexity of the GSA, more running time is required. The overall running and convergence times for 500 iterations were 3079 and 856 s, respectively.

**Figure 14:**
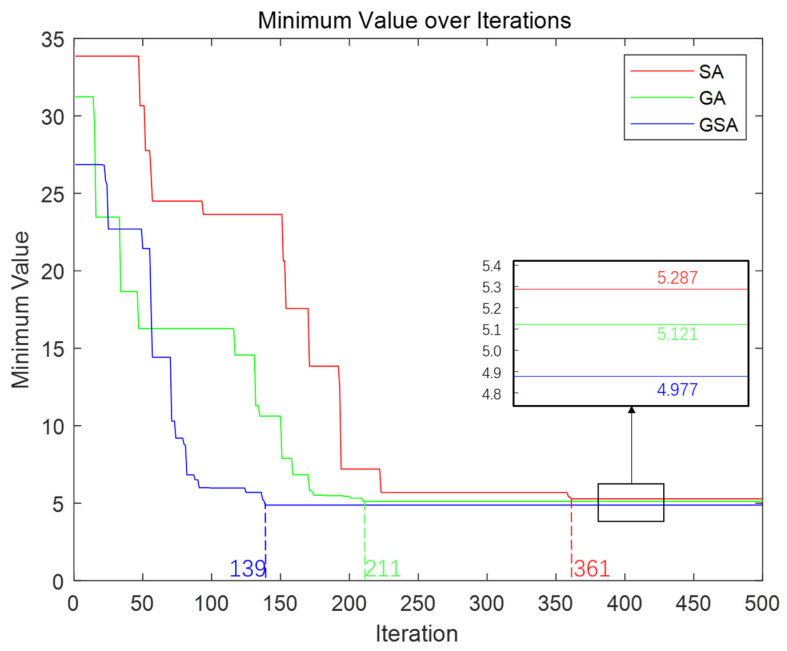
Curves of the number of iterations and the optimal evaluation function values for SA, GA, and GSA.

To compare the performances of the SA, GA, and GSA, we plotted changes in the best error function values with the number of iterations and placed them in the same coordinate system, as shown in Figure 14. Additionally, we recorded detailed information regarding the running time, convergence time, best error function values, and corresponding optimal solutions for each of the three algorithms, as presented in Table 1. Based on these data, the following conclusions were drawn:

(1) Although the grid search method is intuitive, its computation time increases exponentially with the search range and accuracy, making it difficult to bear high-dimensional parameter spaces. In addition, the grid search method can only cover the parameter space within a given range and cannot increase the range of local optimal solutions.
(2) Regarding the calibration data for Channel 1 of the echelle spectrometer studied in this work, the SA algorithm has the shortest running time and convergence time compared to the GA and GSA algorithms when the number of iterations is 500. This is because the SA algorithm only needs to maintain the current and optimal states during the search process and can accept a certain probability of inferior solutions. Therefore, compared to GA and GSA, the SA algorithm requires fewer calculations of function values and fewer individuals, thereby reducing the algorithm’s calculation time and number of iterations.
(3) Although the SA performs well in terms of running time, it may not be as effective as the GA and GSA in discovering global optimal solutions. SA requires 361 iterations to converge, whereas the GA and GSA achieve convergence in 211 and 139 iterations, respectively.
(4) With the same number of iterations, the GSA has the longest running time but the best error function value of 4.977. In contrast, although the SA and GA have shorter running times, their best error function values are 5.287 and 5.121, respectively. Therefore, for high-dimensional and complex optimization problems such as the calibration of an echelle spectrometer, the GSA can attain the optimal error function value, thus achieving higher calibration and measurement accuracy. Hence, it emerges as a superior choice.

**Figure 15:**
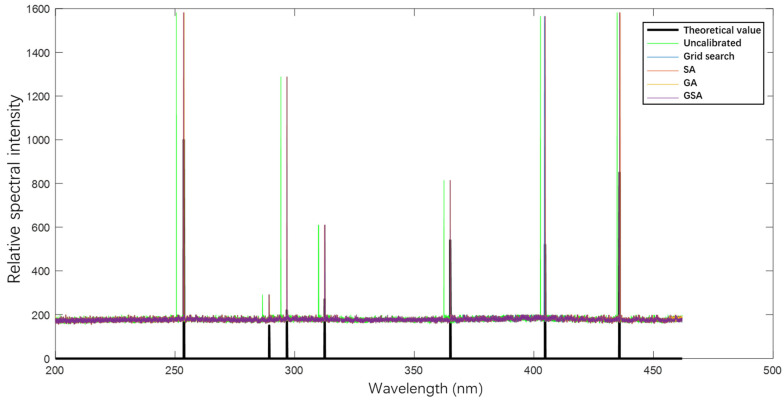
Calibration results of different spectral reduction algorithms of Channel 1.

**Figure 16:**
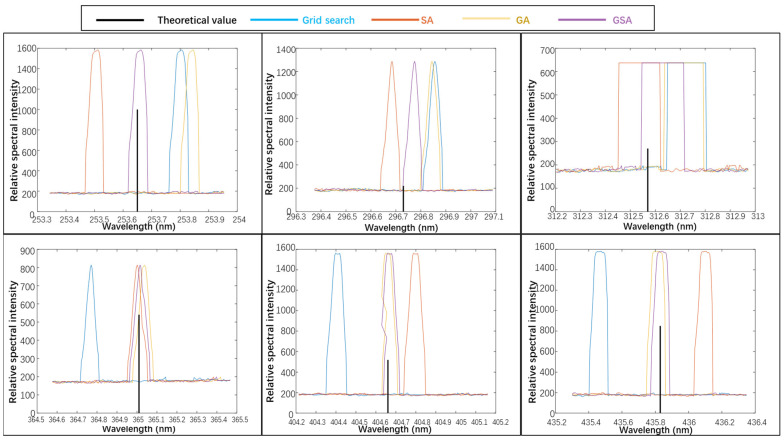
Calibration results of different algorithms for each characteristic wavelength of Channel 1.

To verify the aforementioned analysis, we conducted precise parameter inversion and calibration using different calibration methods on the measured image of the mercury lamp in Channel 1; the results are shown in Figure 15. The spectral curve obtained without parameter inversion and calibration of the spectral model differs significantly from the theoretical values of the characteristic wavelengths of the mercury lamp. The spectral intensity curves calibrated using different inversion algorithms are presented in Figure 16, and the specific error values are given in Table 2.

The average absolute error between the theoretical spectral model and actual instrument wavelength without calibration is 2.03 nm, which cannot meet the requirements of instrument usage. Using the traditional grid search method, SA algorithm, and GA can improve the mean absolute error level to 0.1814 nm, 0.16 nm, and 0.1014 nm, respectively, but these methods fail to satisfy the instrument resolution requirement of 0.1 nm [23]. However, the proposed algorithm can improve the error to 0.0257 nm while meeting the resolution requirements.

**Figure 17:**
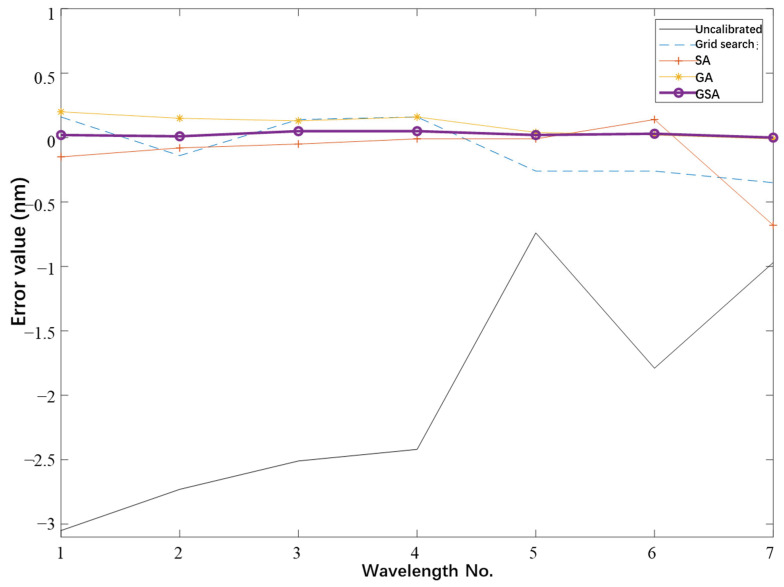
Error values of each characteristic wavelength for different algorithms.

Furthermore, the comparison in Figure 17 reveals that the maximum error of the GSA algorithm among the seven characteristic wavelengths is only 0.05 nm, while those of the grid search method, SA algorithm, and GA are 0.35 nm, 0.68 nm, and 0.2 nm, respectively. These results demonstrate that the proposed algorithm yields excellent spectral restoration performance for a mid-level grating spectrometer to realize high spectral resolution instruments.

## 5. Experiment

After the precise inversion of instrument parameters using the GSA calibration algorithm, we were able to obtain updated and accurate two-dimensional spectra of the echelle spectrometer. To validate the performance of the calibrated echelle spectrometer, we conducted Laser-induced breakdown spectroscopy (LIBS) experiments.

### 5.1. LIBS Principle and Experimental Setup

LIBS is an analytical technique based on atomic emission spectroscopy. It employs high-energy pulsed lasers to ablate samples, creating high-temperature, high-density laser plasma to rapidly analyze material composition and concentration [35]. Compared to traditional analytical methods, LIBS requires no complex pretreatment and can detect materials online and in situ without damaging the sample. Because the technique can analyze gas, liquid, and solid samples, regardless of their physical state, it is widely used in various fields such as petroleum, geology, materials, forensic science, archaeology, metallurgy, environmental monitoring, biomedicine, deep space exploration, and national defense. LIBS technology can remotely and nondestructively analyze materials, qualitatively identify substances, and quantitatively analyze their components with high accuracy and sensitivity. In addition, this technology can achieve non-contact and non-destructive detection, is suitable for online detection in long-distance and harsh environments, and has broad application prospects in the industrial and military fields.

**Figure 18:**
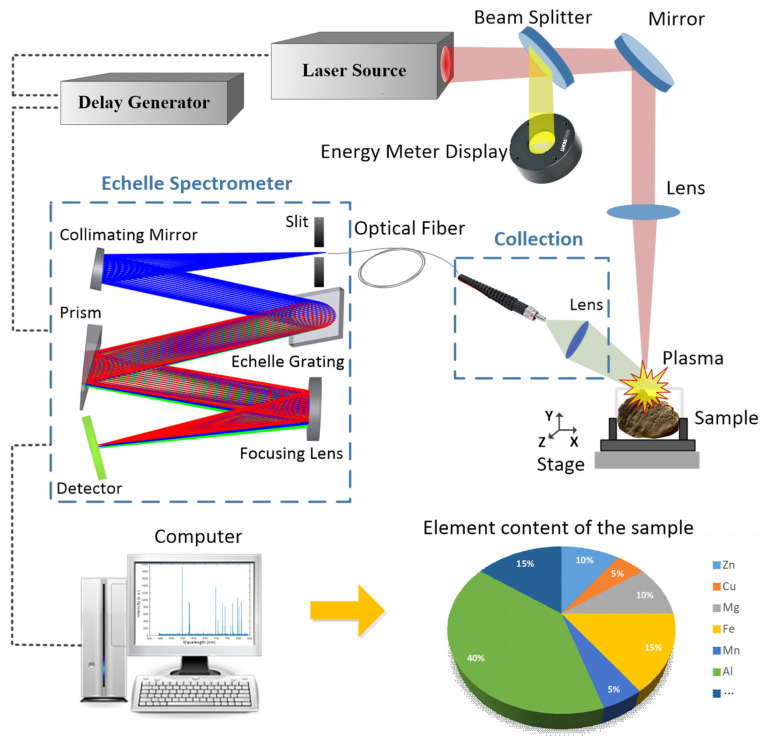
LIBS experimental diagram based on Echelle spectrometer.

A LIBS experimental system was constructed based on the LIBS measurement principle, as shown in Figure 18. The excitation light source is a Nd:YAG nanosecond pulsed laser with a wavelength of 1064 nm, pulse width of ~8 ns (FWHM), and working frequency of 10 Hz. The light beam emitted from the laser was first reflected onto a laser energy meter through a beam splitter to monitor the incident laser energy in real-time. Most of the energy transmitted through the beam splitter was reflected by a 45° mirror to change the direction of the light path and focused on the sample surface through focusing lens L1 (f = 150 mm). The sample was placed on a three-dimensional translation stage to adjust its position precisely. Moreover, the focus point was adjusted slightly below the sample surface to avoid inaccurate measurement results caused by the high-energy laser beam breakdown in the air above the sample surface. We improved the collection efficiency of the plasma radiation spectrum generated after excitation by introducing a positive lens L2 in front of the collecting optical fiber and focusing the light on the front-end face of the optical fiber. The rear face of the optical fiber was connected to the slit of the echelle spectrometer designed in this study. As the laser emits pulses, an external trigger signal is also emitted. The physical process of the plasma changes can be studied by adjusting the relative delay of the spectrometer using a delay-control system. The delay time in this study is based on the moment the laser hits the sample surface.

The spectral information collected by the echelle spectrometer was recorded using a detector based on the calibrated spectral model. Compared with the NIST database [36], the emission spectrum lines of different elements can be determined, and qualitative and quantitative analyses of the elements can be achieved.

### 5.2. Experimental Results and Analysis

**Figure 19:**
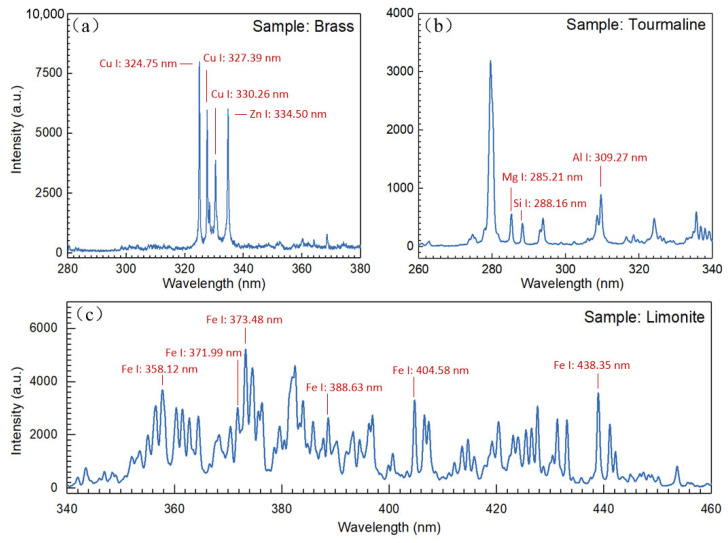
LIBS spectra and elemental characteristic peaks of (**a**) Brass, (**b**) Tourmaline, and (**c**) Hematite detected by echelle spectrometer after calibration using the GSA algorithm.

The experimental principle of LIBS in Figure 18 was employed to conduct experiments on various types of rock and metal samples (brass, tourmaline, and hematite). The plasma was excited by a pulsed laser beam with a pulse energy of 33 mJ, and the spectral information was obtained using a high-time-resolution intermediate-dispersion grating spectrometer. The integration time for a single exposure to the instrument was set to 10 ns. By comparison with the NIST database, the emission spectral lines of the main elements in the different samples were determined. After analysis, the characteristic spectral lines of five metal elements, Cu I:324.75, Cu I:327.39, Cu I:330.26, Zn I:334.50, Mg I:285.21, Si I:288.16, Al I:309.27, Fe I:358.12, Fe I:371.99, Fe I:373.48, Fe I:388.63, Fe I:404.58, and Fe I:438.85 nm, were identified and labeled in the corresponding spectral curves in Figure 19.

**Figure 20:**
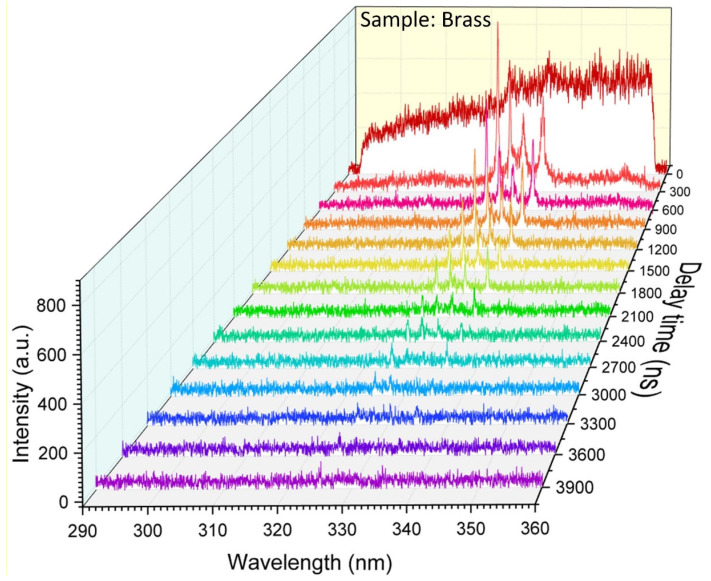
Time-resolved LIBS spectra of brass samples captured by the echelle spectrometer.

A high-temporal resolution echelle spectrometer detected the plasma temporal resolution spectrum to validate the temporal resolution of the instrument and provide conditions for in-depth research on the plasma evolution processes. Figure 20 shows the temporal resolution spectrum of the brass sample. From the moment of laser breakdown on the sample surface, taken as the zero moment, we considered a delay time step of 300 ns and a termination time of 3900 ns; each sampling integration time was 10 ns. The results of the plasma spectrum corresponding to different delay times showed that the intensity of the continuous spectral signal first increased and then decreased rapidly, followed by an overall trend of slow enhancement and decay of the separated spectral signal with the delay time. In laser-induced plasma, the separated and continuous spectral signals are caused by stimulated radiation (bond-bond transition) and bremsstrahlung radiation (free-free transition), respectively. Because stimulated radiation occurs later than bremsstrahlung radiation, different spectral radiation signals exhibit different temporal evolution patterns, corresponding to different window times. The evolution of element types and content can be determined by studying the changes in the spectral lines with time.

## 6. Conclusions

We have successfully developed a compact, asymmetric three-channel echelle spectrometer with remarkable high-spectral resolution capabilities. By establishing a comprehensive theoretical spectral model utilizing the two-dimensional coordinates of spot positions associated with each wavelength, we have devised a guiding framework for the calibration process, facilitating the attainment of the desired spectral resolution.

Furthermore, we introduced a groundbreaking method for precisely calibrating echelle spectrometers based on the principles of parameter inversion. The proposed GSA algorithm combines the global search capabilities of the GA and the local search abilities of SA for the simultaneous optimization of multiple parameters to enhance calibration accuracy. Our research delved into the inherent complexity of the two-dimensional nonlinear echelle spectrogram and employed various optimization techniques, such as grid research, SA, GA, and GSA algorithm, to accurately invert spectrogram parameters. Compared with the traditional grid search method, the proposed GSA algorithm reduces the error function by 22.8% and convergence time by 2.16 times while improving the calibration accuracy by 7.05 times. Furthermore, the average spectral accuracy error of the low-pressure mercury lamp obtained after inverting the key parameters was only 0.0257 nm. Finally, the developed echelle spectrometer in a LIBS experiment demonstrated exceptional spectral resolution and better than 10-ns time-resolved capability, positioning it as a valuable tool for various scientific applications.

However, if the number of parameters to be inverted increases, the algorithm’s dimensionality will also increase, leading to longer computation times. It is important to acknowledge that higher-dimensional parameter inversions require additional computational resources and may result in longer optimization processes. Careful consideration should be given to optimizing the algorithm’s efficiency and scalability to handle such scenarios effectively. Future research can focus on developing strategies to address the computational challenges associated with higher-dimensional parameter inversions, ensuring reasonable execution times while maintaining spectral precision.

## Figures and Tables

**Table 1:** Comparison of results among four different algorithms.

Indicator	Grid Research	SA	GA	GSA
Number of iterations	-	500	500	500
Run time	1853 s	756 s	1578 s	3079 s
Number of convergences	-	361	211	139
Optimal solution of the incident angle of the grating	45.84°	46.170°	46.339°	46.2317°
Optimal solution of the deflection angle of the grating	10.24°	10.225°	10.217°	10.1775°
Optimal error function value $(\sum_{i=1}^{n}\sqrt{\frac{a{\left(\Delta x_{i}\right)}^{2}+b{\left(\Delta y_{i}\right)}^{2}}{2}}$)	6.443	5.287	5.121	4.977
Convergence time	-	546 s	666 s	856 s

**Table 2:** Different algorithms for mercury lamp calibration wavelength and error.

No.	Wavelength (nm)	Uncalibrated	Grid Search	SA	GA	GSA
1	253.65	250.60	253.81	253.50	253.85	253.67
2	289.36	286.63	289.22	289.28	289.51	289.37
3	296.73	294.22	296.87	296.68	296.86	296.78
4	312.57	310.15	312.73	312.56	312.73	312.62
5	365.01	364.27	364.75	365.00	365.05	365.03
6	404.66	402.87	404.40	404.80	404.68	404.69
7	435.83	434.86	435.48	435.15	435.82	435.83
Mean absolute error (nm)		2.03	0.1814	0.16	0.1014	0.0257

## Data Availability

The data presented in this study are available from the corresponding author upon request.
